# Quantitative ultra-micro angiography assessment of dynamic cerebral microperfusion patterns by gestational age in neonates: a prospective observational cohort study

**DOI:** 10.3389/fped.2026.1775558

**Published:** 2026-05-22

**Authors:** Yahui Zhang, Yanxia You, Yinxing Hu, Rui Li, Jianqiu Huang, Yunfeng Liu, Tongyan Han

**Affiliations:** 1 Department of Pediatrics, Peking University Third Hospital, Beijing, China; 2 Department of Ultrasonography, Peking University Third Hospital, Beijing, China

**Keywords:** cerebral perfusion, color pixel percentage, neonatal microcirculation, preterm brain development, ultra-micro angiography

## Abstract

**Introduction:**

We aimed to establish normative developmental patterns of cerebral microperfusion using ultra-micro angiography and evaluate regional microvascular differences in neonates across gestational ages.

**Methods:**

This prospective observational cohort study enrolled 115 neonates from a single-center neonatal intensive care unit or neonatal ward at Peking University Third Hospital (2023–2024). The patients were stratified by postmenstrual age into extremely/very preterm (*n* = 30), moderate/late preterm (*n* = 23), and term (*n* = 62) groups, excluding those with major anomalies/hemodynamic instability. Ultra-micro angiography was performed via the anterior/sphenoid fontanelles during quiet sleep (3–14 days postnatal). Regional color pixel percentage (CPP) and large-vessel hemodynamics [peak systolic velocity [PSV]/end-diastolic velocity [EDV]/ resistance index [RI]] were measured.

**Results:**

Ultra-micro angiography revealed distinct postmenstrual age-dependent microvascular perfusion patterns, with cortical and white matter CPP demonstrating progressive increase across postmenstrual age groups (extremely preterm vs. term infants: frontal lobe CPP 22.97 [IQR 20.12–28.92] vs. 47.57 [40.07–55.93]; parietal lobe CPP 25.09 [20.96–29.94] vs. 47.69 [38.93–55.97]), while basal ganglia CPP remained stable [32.96 [30.64–35.02] vs. 33.77 [31.52–37.46]]. Midline regions consistently exhibited the highest perfusion across all ages [term infants: 66.26 (61.33–70.62)]. Concurrent macrovascular assessment showed that anterior/middle cerebral artery peak systolic and end-diastolic velocities increased with maturation, although resistance indices maintained stability.

**Conclusion:**

Ultra-micro angiography-derived CPP quantifies gestational age-dependent microvascular maturation, revealing distinct regional perfusion patterns. Differences in the progression of CPP in various brain regions may serve as a biomarker for risk stratification of neurodevelopment in premature infants.

## 1 Introduction

Brain development is a complex, continuous process that begins in the embryonic period and extends for years after birth (1). The brain accounts for less than 3% of an adult's body weight; however, it receives 15% of the blood supply and consumes 25% of the body's total oxygen (2). After birth, newborns, especially premature infants, have limited self-regulation ability. Their respiratory and circulatory transitions cause significant hemodynamic fluctuations, which are highly likely to interfere with cerebral blood supply and lead to severe brain injury (3–5). Moreover, even when systemic hemodynamics appear normal, cerebral microcirculation may be impaired (5–8). Such microcirculatory imbalance can lead to misjudgment of the clinical condition and the use of ineffective or harmful treatments, particularly in critically ill neonates (8). Therefore, monitoring neonatal cerebral microcirculation to assess tissue perfusion and oxygen delivery is essential for accurate clinical assessment, optimizing treatment, and predicting neurological outcomes.

However, for newborns, particularly premature infants, restrictions against radiation exposure, application of contrast agents and sedatives, and limited examination sites exist. Therefore, a feasible, convenient, valid, and widely recognized evaluation index for cerebral microcirculation perfusion needs to be established (9–12). Head ultrasound serves as a first-line method for evaluating neonatal brains (3, 5, 13–15) because it is noninvasive, convenient, safe, and reproducible and provides high-resolution imaging. The addition of color Doppler flow imaging further enhances its utility, particularly in the assessment of cerebral vessels within the brain parenchyma (14). More recently, ultra-micro angiography (UMA) has emerged as a technique that can precisely monitor micro-blood flow in localized intracranial regions (3, 11, 13–18). Depending on the ultrasound equipment manufacturer, this technique is also referred to as microvascular imaging, superb microvascular imaging, or microvascular flow imaging, among other names. However, studies on UMA for brain development in preterm and neonatal populations remain limited, and the lack of standardized quantitative reference ranges restricts its broader clinical application (13, 14).

At present, several critical gaps persist, such as (1) whether brain microperfusion patterns differ across brain regions; (2) how neonatal cerebral microperfusion is associated with gestational age; and (3) the lack of a quantifiable, noninvasive, and feasible method for assessing microcirculatory perfusion in different regions of the neonatal brain. Therefore, cerebral microcirculation patterns are hypothesized to vary across brain regions and to be related to gestational age. Color pixel percentage (CPP) measurement using UMA may serve as a reproducible biomarker for evaluating neonatal cerebral microcirculation. By testing these hypotheses, this study aimed to establish normative baseline values for neonatal brain microcirculation, particularly in preterm infants, and explore the potential clinical application of UMA in neurodevelopmental risk stratification.

## 2 Materials and methods

### 2.1 Study design

In this prospective, observational, single-center study, changes in cerebral microvascular flow were evaluated in neonates across different postmenstrual ages (PMAs), and regional differences within the brain were compared.

### 2.2 Participants

Neonates admitted to the neonatal intensive care unit (NICU) or neonatal ward of Peking University Third Hospital between May 1, 2023, and April 30, 2024, were enrolled in the study.

Exclusion criteria were: (1) major congenital/chromosomal anomalies; (2) significant neurological pathology (intraventricular hemorrhage > grade II, stroke, hypoxic–ischemic encephalopathy, white matter damage, periventricular leukomalacia, central nervous system infection); (3) hemodynamic instability [shock, vasopressor use, hemodynamically significant patent ductus arteriosus (hsPDA)]; (4) major organ dysfunction (respiratory, renal, gastrointestinal, hematologic); and (5) technical limitations (sedation, poor acoustic windows). hsPDA definition is based on a previous study (19) whereas invasive ventilation parameters included mean arterial pressure (MAP) > 8 mmHg/fraction of inspired oxygen (FiO2) > 0.30.

**Figure 1:**
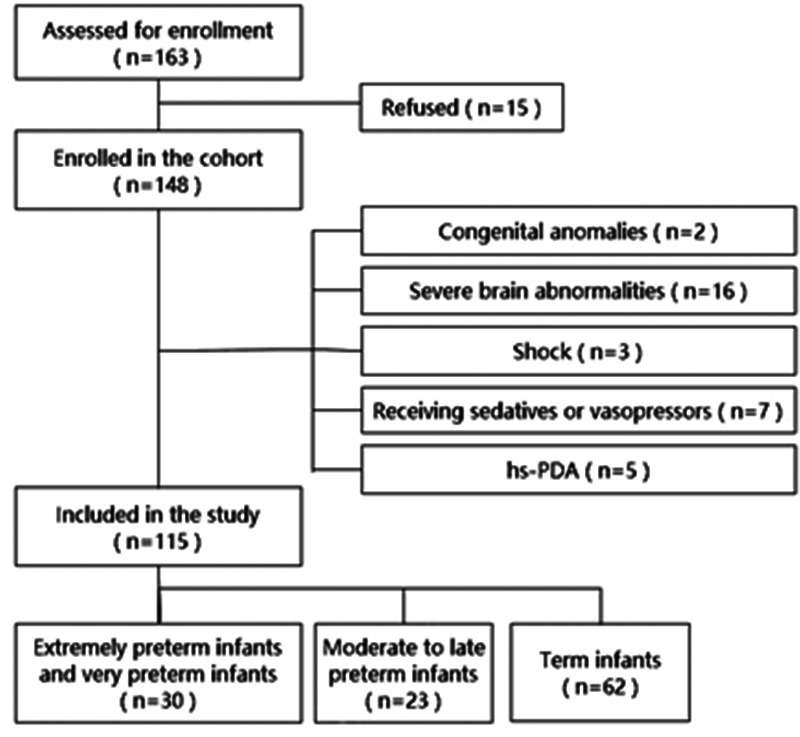
Study enrollment and final cohort allocation.

A total of 115 newborns met the inclusion criteria and were divided into the extremely preterm and preterm group, the middle and late preterm group, and the full-term group according to the postmenstrual age (PMA). The screening process is illustrated in Figure 1.

Owing to the technical nature of neonatal cerebral perfusion imaging and the vulnerable status of participants, formal PPI was not integrated into the study design phase. However, guardians of enrolled neonates provided informed consent after receiving detailed explanations of the research objectives and procedures. To ensure transparency, study findings will be disseminated to participating families through lay-language summaries and hospital-based educational sessions.

### 2.3 Instruments, inspection methods, image acquisition, and data measurement and collection

#### 2.3.1 Instruments and parameters

Neonatal cranial ultrasound assessments were conducted using a Mindray Resona R9Q system equipped with both C11-3U (3.0–11.0 MHz) and L15-3U (3.0–15.0 MHz) linear array probes for ultra-micro angiography (UMA) imaging, with UMA parameters set at 50 dB gray-scale gain, 40 dB flow gain, and maintained within safe operating limits (MI 0.4–0.8, TI 0.01).

#### 2.3.2 Inspection methods

All infants received supine transcranial ultrasound between 3 and 14 postnatal days during quiet sleep, with head neutral and fontanelles exposed. Standard gray-scale and Doppler imaging assessed anterior/middle cerebral arteries (PSV, EDV, and RI averaged over 3 cycles). The UMA mode was applied to visualize parenchymal microvessels and quantified regional perfusion via color pixel percentage (CPP).

#### 2.3.3 Image acquisition

In accordance with the recommendations of the American Association of Ultrasound in Medicine, the right side of the participant was displayed on the left side of the image during coronal scanning, and the forehead was displayed on the left side during sagittal scanning. Image acquisition was performed using a C11-3U micro-convex probe to obtain gray-scale sonograms and CDFI images of the target area, with the blood flow velocity scale set to a minimum value of 2.9 cm/s. The region of interest (ROI) within the target area was then selected. UMA imaging mode was subsequently applied to acquire images and measure the CPP, with the blood flow velocity scale set to a minimum value of 2.5 cm/s.

#### 2.3.4 Measurement of CPP

Using UMA imaging, we placed 1 cm^2^ ROIs (0.79 cm^2^ area) over five brain regions (frontal/parietal cortex, basal ganglia, midline junction, and white matter) to quantify the microvascular blood flow (3, 15). The system automatically calculated CPP as a flow richness index. Triplicate measurements per site were averaged to reduce error.

#### 2.3.5 Anterior cerebral artery and middle cerebral artery measurement

CDFI visualized ACA/MCA and their major branches, while pulsed-wave Doppler measured PSV, EDV, and RI to assess neonatal intracranial hemodynamics.

#### 2.3.6 Other data collection

The following data were obtained from the hospital's electronic data management system to ensure accuracy and completeness: (1) maternal factors (complications, delivery mode); (2) perinatal indicators (Apgar score, resuscitation needs); (3) neonatal parameters (GA, growth percentiles, critical laboratory results); and (4) hemodynamic status (BP, SpO^2^, respiratory support).

#### 2.3.7 Interobserver consistency assessment

Two trained physicians (A and B) independently conducted three neonatal cerebral microvascular flow images and calculated their mean values, blinded to each other's results. Interobserver consistency was assessed; discrepancies were resolved by joint remeasurement and consensus.

### 2.4 Data analysis

Analyses were performed using SPSS for Windows, version 26.0 (IBM Corp., Armonk, NY, USA) and R package's strucchange. Normally distributed data are expressed as mean ± standard deviation [SD] (ANOVA with LSD post-hoc); non-parametric data, as median (IQR) (Kruskal–Wallis with Bonferroni adjustment). Categorical variables were compared using *χ*² tests with Bonferroni correction (significance was set at *P* < 0.05).

For hemispheric correlation trends, piecewise linear regression identified inflection points via least squares (*F*-test significance). Bootstrap methods determined 95% confidence intervals (CIs) for inflection locations (R implementation). All tests used *α* = 0.05 threshold.

### 2.5 Ethical approval

This study was approved by the Ethics Committee of Peking University Third Hospital (No. M2022211) and complied with the Declaration of Helsinki and local regulations. All participants' guardians provided written consent. Trial registration number: NCT05761548.

## 3 Results

### 3.1 Comparison of baseline features between groups

Among the enrolled participants, 51.50% were female. No significant differences were observed among the three groups in the proportion of small-for-gestational-age infants, rate of cesarean delivery, rate of multiple pregnancies, incidence of maternal complications during pregnancy, or proportion of *in vitro* fertilization. Similarly, no significant differences were found in baseline respiratory rate or Apgar scores at birth among the groups.

All patients exhibited vital signs within the normal range on the day of examination. Significant differences were observed in body weight, basal heart rate, and blood pressure among the three groups (all *P* < 0.05). Basal heart rate was lowest in full-term infants, whereas blood pressure was the lowest in the extremely preterm and very preterm groups. Body weight was the lowest in the extremely preterm and very preterm groups and highest in the full-term group (Table 1). Despite these differences, heart rate, respiratory rate, and blood pressure remained within normal limits in all enrolled neonates on the day of examination.

**Table 1:** Comparison of general characteristics, perinatal factors, vital signs and parameters of great cerebral arteries among the three groups of children.

Variable	Full group	Extremely preterm and very preterm infants	Moderate-to-late preterm infants	Term birth	*P*
N	115	30	23	62	
Sex (females) *n* (%)	59 (51.50)	16 (53.33)	18 (78.26)	25 (40.32)	0.112
Corrected gestational age (days)	262 (223–275)	203 (192–213)	240 (232–245.5)*	274 (267–285.5)^#^^,^^+^	<0.001
Weight on the day of examination (g)	2,780 (1,460–3,350)	970 (810–1,190)	2,350 (1,850–2,620)*	3,300 (3,010–3,580)^#^^,^^+^	<0.001
SGA *n* (%)	12 (10.43)	4 (13.33)	2 (8.70)	6 (9.68%)	0.826
Cesarean section *n* (%)	69 (60.00)	19 (63.33)	17 (73.91)	33 (53.23%)	0.132
Multiple pregnancy *n* (%)	37 (32.17)	9 (30.00)	11 (47.83)	17 (27.42%)	0.193
Assisted reproduction *n* (%)	33 (28.70)	9 (30.01)	10 (43.48)	14 (22.58%)	0.214
Gestational hypertension *n* (%)	23 (20.00)	9 (30.02)	5 (21.74)	9 (14.52%)	0.077
Gestational diabetes *n* (%)	22 (19.13)	4 (13.33)	2 (8.70)	16 (25.81%)	0.341
Apgar score 1 min	10 (8–10)	10 (6.75–10)	10 (7.5–10)	10 (9.25–10)	0.134
Apgar score for 5 min	10 (10–10)	10 (8.75–10)	10 (10–10)	10 (10–10)	0.315
Apgar score 10 min	10 (10–10)	10 (9–10)	10 (10–10)	10 (10–10)	0.758
Heart rate (beats/min)	136.08 ± 18.93	148.37 ± 18.92	144.43 ± 18.47	127.03 ± 13.89^#^^,^^+^	<0.001
R (times/min)	39.00 (34.50–42.00)	40.00 (36.00–43.00)	41.00 (35.50–42.50)	39.00 (32.00–41.00)	0.129
SBP (mmHg)	76.00 (67.00–81.00)	64.00 (57.00–70.00)	75.00 (67.50–79.00)*	79.00 (74.00–84.00)^#^	<0.001
DBP (mmHg)	42.00 (37.50–46.00)	35.00 (29.00–38.00)	42.00 (38.00–45.00)*	45.00 (42.00–49.00)^#^	<0.001
MAP (mmHg)	53.00 (48.50–57.00)	44.00 (39.00–49.00)	53.00 (48.50–56.50)*	53.00 (53.00–59.00)^#^	<0.001
ACA PSV (cm/s)	37.63 ± 12.83	23.61 ± 5.61	33.60 ± 3.86	45.90 ± 10.72^#^^,^^+^	<0.001
ACA EDV (cm/s)	9.95 ± 3.53	6.50 ± 1.80	9.27 ± 2.00	11.87 ± 3.25^#^^,^^+^	0.008
ACA RI	0.74 (0.69–0.78)	0.72 (0.67–0.77)	0.74 (0.69–0.76)	0.75 (0.69–0.79)	0.266
MCA PSV (cm/s)	50.22 (39.55–60.39)	36.54 (26.28–39.61)	43.44 (39.17–47.26)	59.39 (53.57–71.61)^#^^,^^+^	<0.001
MCA EDV (cm/s)	15.65 ± 5.90	9.87 ± 2.59	13.50 ± 2.69	19.29 ± 5.26^#^^,^^+^	0.013
MCA RI	0.70 ± 0.07	0.71 ± 0.07	0.69 ± 0.06	0.69 ± 0.07	0.621

* Significant differences are observed among extremely preterm infants, very preterm infants, and moderate-to-late preterm infants.

# Significant differences are observed among extremely preterm infants, very preterm infants, and term infants.

+ Significant differences are observed between moderate-to-late preterm group and term group.

### 3.2 Comparison of ACA and MCA parameters

Significant differences in the PSV and EDV of the ACA and MCA were observed among the three groups (all *P* < 0.05). Pairwise comparisons indicated that ACA PSV, ACA EDV, and MCA EDV were the lowest in the extremely preterm and very preterm groups and highest in the full-term group. MCA PSV was highest in the full-term group, with no significant difference between the other two groups. The RI of both ACA and MCA did not differ significantly among all groups, suggesting that blood flow in the major cerebral arteries remained within normal physiological limits and that overall cerebral perfusion was stable (Table 1).

### 3.3 Comparison of microvascular perfusion among different PMA groups

**Figure 2:**
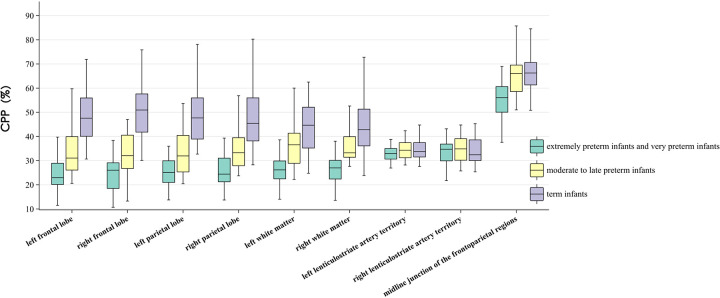
Regional cerebral blood flow patterns across gestational ages.

No significant differences were observed in CPP within the bilateral basal ganglia among the three groups. By contrast, significant differences were noted in the frontal lobe, parietal lobe, white matter, and midline regions (all *P* < 0.05). CPP values in the parietal lobe and white matter were the lowest in the extremely preterm and very preterm groups and highest in full-term group. Bilateral frontal lobe CPP was the highest in the full-term group, with no significant difference observed between the other two groups. Midline CPP was the lowest in the extremely preterm and very preterm groups, with no significant difference observed between the remaining two groups (Table 2 and Figure 2).

**Table 2:** Comparison of CPP at each site among the three groups [median (Q1–Q3), range (min-max)].

Brain region	Full group	Extremely preterm and very preterm infants	Moderate-to-late preterm infants	Term babies	*P*
Midline area	63.93 (57.21–68.92)	56.07 (50.05–60.64)	66.04 (58.61–69.53)*	66.26 (61.33–70.62)^#^	<0.001
	(37.57–87.13)	(37.57–68.98)	(51.02–85.75)	(50.81–87.13)	
Left frontal lobe	38.04 (27.54–49.67)	22.97 (20.12–28.92)	31.06 (26.09–39.92)	47.57 (40.07–55.93)^#^^,^^+^	<0.001
	(11.47–71.89)	(11.49–39.73)	(20.56–59.74)	(30.66–71.89)	
Right frontal lobe	38.18 (29.09–51.49)	26.03 (18.50–29.11)	32.08 (26.74–40.53)	50.96 (41.80–57.62)^#^^,^^+^	<0.001
	(10.70–78.12)	(10.70–38.32)	(13.27–47.04)	(30.08–75.86)	
Left parietal lobe	37.71 (28.02–51.18)	25.09 (20.96–29.94)	31.95 (25.33–40.44)*	47.69 (38.93–55.97)^#^^,^^+^	<0.001
	(13.74–78.12)	(13.74–35.97)	(20.43–53.66)	(32.71–78.12)	
Right parietal lobe	37.46 (28.39–51.34)	24.42 (21.26–31.02)	33.25 (27.91–39.50)*	45.45 (38.17–55.98)^#^^,^^+^	<0.001
	(13.71–80.27)	(13.71–39.33)	(23.73–58.69)	(28.27–80.27)	
Left white matter	36.07 (29.20–49.21)	26.18 (22.48–29.87)	36.55 (28.90–41.35)*	44.67 (35.22–52.11)^#^^,^^+^	<0.001
	(14.03–62.52)	(14.03–38.62)	(22.21–60.92)	(24.78–62.52)	
Right white matter	36.47 (30.36–44.79)	27.00 (22.40–30.12)	33.22 (31.42–39.90)*	42.84 (36.12–51.30)^#^^,^^+^	<0.001
	(13.54–72.77)	(13.54–38.05)	(27.61–55.97)	(23.84–72.77)	
Left lenticulostriate artery	33.54 (31.10–37.11)	32.96 (30.64–35.02)	34.28 (31.23–37.43)	33.77 (31.52–37.46)	0.216
	(26.93–44.75)	(26.93–38.80)	(28.19–42.39)	(27.60–44.75)	
Right lenticulostriate artery	33.75 (30.01–37.96)	34.73 (29.92–36.84)	34.86 (30.17–39.08)	32.41 (30.01–38.61)	0.845
	(21.72–45.30)	(21.72–43.16)	(25.74–44.76)	(25.37–45.30)	

* Significant differences are observed among the extremely preterm, very preterm, and moderate-to-late preterm groups.

# Significant differences are observed among extremely preterm infants, very preterm infants, and term infants.

+ Significant differences are observed between the moderate-to-late preterm group and term group.

**Figure 3:**
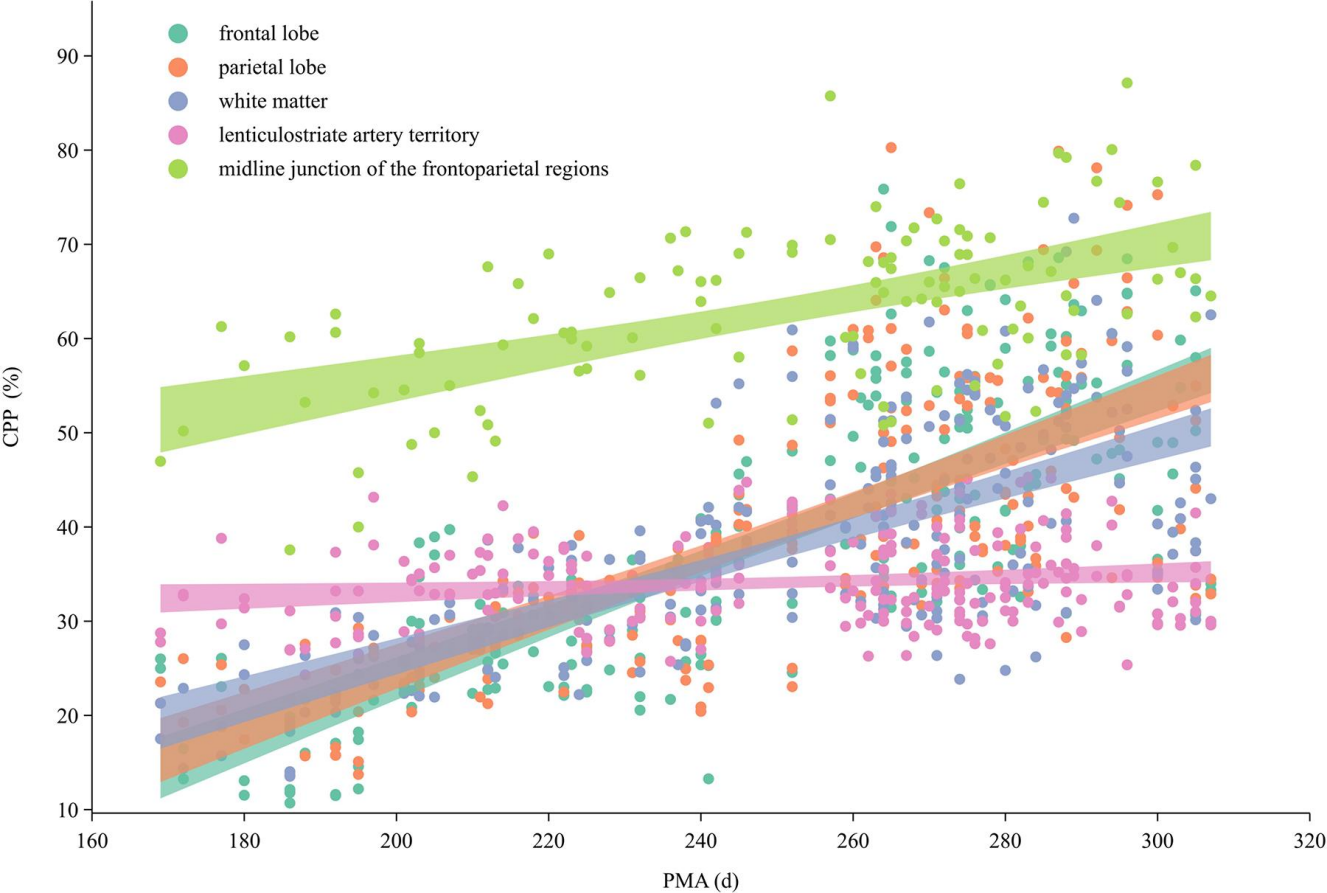
Choroid plexus perfusion dynamics across postmenstrual ages.

In this study, scatter plots were used to illustrate the relationship between CPP values in each brain region and gestational age distribution (Figure 3). Coefficient of determination (*R*^2^) values were then calculated for the fitted curves. For the bilateral frontal lobes, parietal lobes, and white matter, the logarithmic regression model provided the best fit, with *R*^2^ values of 0.539, 0.562, 0.530, 0.463, 0.506, and 0.476, respectively (all *P* < 0.05), indicating a positive correlation with PMA. The results demonstrated that CPP values in the cerebral cortex and white matter increased steadily with PMA, with no definite inflection points observed.

### 3.4 Comparison of microperfusion in different brain regions

In this study, significant differences were found in CPP values across brain regions within the three PMA groups, with the distribution patterns varying by gestational age. In the extremely preterm and very preterm groups, CPP in the midline and basal ganglia regions was significantly higher than that in the frontal lobe, parietal lobe, and white matter (all *P* < 0.05), with midline CPP also significantly higher than that in the basal ganglia CPP (*P* = 0.027). In the moderate-to-late preterm group, only CPP in the midline region was significantly higher than that in other regions (all *P* < 0.05). Among full-term infants, midline CPP remained significantly higher than that in other regions, whereas CPP in the frontal lobe, parietal lobe, and white matter was higher than that in the bilateral basal ganglia (all *P* < 0.05) (Figure 3). No significant differences were found in bilateral CPP at the same sites within each PMA group.

## 4 Discussion

This study demonstrated significant increases in CPP of the cerebral cortex and white matter in neonates with advancing PMA but showed no clear correlation with that in the basal ganglia. Notable differences in midline CPP were observed among extremely, very, and moderate-to-late preterm groups, reflecting rapid microvascular maturation. However frontal lobe CPP only differed significantly between moderate-to-late preterm and term infants. These findings are consistent with established models of brain development (20). As the brain undergoes structural and functional maturation, metabolic demand and blood perfusion diverge across different regions (21, 22). The basal ganglia's stable CPP across PMA groups suggests early functional maturity. This is consistent with its fundamental role in neurodevelopment, although vascular development appears to plateau during the third trimester (21, 23–26). Discrepancies of our results with those of prior studies may stem from methodological or population differences (27).

Cortical CPP showed significant postnatal increases in the parietal and frontal lobes, contrasting with the stable basal ganglia perfusion—highlighting regional brain maturation differences. The perinatal period marks the peak human brain growth, transitioning from neuronal migration (early gestation) to glial proliferation and myelination (third trimester) (28). Around 24 weeks, vascular dominance shifts from the basal ganglia to the cortex, with cortical vessels rapidly maturing through term alongside dramatic cortical expansion (24, 29, 30). Synaptic development drives metabolic shifts toward aerobic pathways, elevating perfusion. Our observed frontal CPP surge reflects both late maturation of higher cognitive functions and their growing metabolic demands (31).

This study revealed consistently higher CPP in the central sulcus compared to other brain regions across all gestational ages, with significantly elevated perfusion in moderate-to-late preterm and term infants vs. their more premature counterparts. As a critical hub integrating motor (Brodmann area 4) and sensory functions (areas 3/1/2), this region's dominant vascular development during late gestation supports its sustained hyperperfusion—detectable via UMA—to meet high metabolic demands and prevent hypoxic-ischemic injury (13).

This study found increasing white matter CPP with gestational age. While critical for neural conduction, white matter's low vascularity makes it hypoxia-sensitive, though vascular maturation improves in late preterm-to-term infants (32). No CPP differences emerged between white matter and frontoparietal cortex, possibly due to sample size or monitoring site limitations—warranting larger studies for confirmation.

Scatter plots revealed linear CPP-PMA correlations in the frontal/parietal lobes, white matter, and central regions (R² analysis). Although intra-group CPP variability reflected individual perfusion differences, no hemispheric asymmetry emerged, possibly due to the participants' early neonatal status (50% preterm), preceding functional lateralization (25, 33).

Extremely preterm infants (<32 weeks GA) show elevated risks for neurodevelopmental disorders and subtle cognitive deficits (34). This study found lower frontal/parietal/white matter CPP vs. basal ganglia—consistent with earlier subcortical maturation findings (active vascular proliferation occurs in caudate/thalamic regions at 24–28 weeks) (26, 32). Conversely, cortical/white matter CPP remained low, due to the immature vasculature and autoregulation, explaining periventricular white matter's clinical vulnerability (e.g., frequent softening) (4). CPP quantitatively captures these regional perfusion disparities, aiding early hypoxia risk detection.

School-age data reveal that late preterm infants face higher neonatal mortality and 36% show elevated developmental delay risk compared to that in term infants, confirming preterm birth's lasting neurological impact (35). Our study shows that moderate-to-late preterm infants have higher cortical/white matter CPP than extremely preterm ones, reflecting normal neurodevelopment when cortical volume reaches 53% in term infants and myelination accelerates near term. However, premature delivery disrupts this critical maturation phase (36). Thus, we recommend routine UMA/CPP monitoring (especially cortex/white matter) to assess microcirculation and enable early neurodevelopmental risk detection.

Term infants showed higher cortical/white matter CPP compared to other regions—consistent with known cortical microvascular density and metabolic demands (21, 37). Although frontoparietal-white matter CPP differences existed, these lacked significance, potentially owing to sample size constraints or region-specific measurement selection.

While prior studies demonstrated progressive fetal intracranial vasodilation and declining vascular resistance during the third trimester (38), we found stable ACA/MCA RI values across neonatal age groups. This discrepancy likely reflects physiological transitions, since intrauterine development requires decreasing resistance to support rapid brain growth, whereas postnatal oxygenation triggers autoregulatory vasoconstriction to prevent hyperperfusion stress (38). Thus, postnatal RI stabilization balances oxygen delivery with metabolic protection.

Combining UMA with CPP quantification provides unprecedented insights into neonatal brain perfusion dynamics, revealing maturation-dependent regional variations (macroscopic ↔ microscopic correlations). Our data establish gestational-age-specific perfusion patterns reflecting developmental priorities—serving both as a diagnostic baseline for perfusion monitoring and enabling early detection of injury risks. This paradigm may ultimately optimize neuroprotective interventions, improving both acute care precision and long-term neurodevelopmental trajectories.

While establishing neonatal cerebral microvascular perfusion references, this study acknowledges three key constraints: (1) limited sample size limiting the generalizability, (2) inherent perfusion dynamism potentially masked by the single-timepoint measurements, and (3) operator-dependent region selection introducing interobserver variability. These factors warrant consideration when interpreting the results and designing future validation studies.

Therefore, future research should enhance the reliability and consistency of measurements by establishing standardized operating procedures, unified measurement sites, and standardized interpretation protocols (39). Furthermore, larger sample sizes are needed to establish more precise reference ranges for CPP and neonatal microvascular perfusion. Such data would provide a quantitative foundation for characterizing normal developmental patterns and identifying abnormal cerebral perfusion patterns. Previous studies have suggested that in various neurological disorders, microcirculatory alterations often precede changes in large vessels and tissue perfusion, and these processes do not occur synchronously (16, 17, 40). Accordingly, UMA may have broad clinical applicability in pathological conditions. Future investigations should explore the correlation between microvascular perfusion and long-term neurodevelopmental outcomes, thereby informing clinical decision-making.

Based on these findings, several recommendations can be made. First, UMA is a valuable modality for monitoring intracranial microcirculation in neonates, particularly in preterm and high-risk infants. The timing of assessments should be individualized according to gestational age and specific clinical conditions. Second, interpretation of monitoring results should consider both gestational age and the regional characteristics of microvascular perfusion to ensure comprehensive analysis and accurate clinical judgment. Ultimately, precise interpretation and individualized management are expected to enhance the neurodevelopmental outcomes in newborns.

## 5 Conclusions

This study, which employed UMA technology to delineate gestational age-dependent perfusion patterns across neonatal brain regions, established the first quantitative microcirculatory reference framework. Cortical and white matter CPP increased significantly with gestational age (except in the basal ganglia), while the central sulcus consistently demonstrated the highest perfusion—likely reflecting its elevated metabolic demands during development. These region-specific patterns provide new insights into functional neurovascular maturation. As a sensitive, reproducible bedside tool, UMA offers clinically actionable advantages, including precise microcirculatory mapping that enables early detection of perfusion abnormalities and optimization of neuroprotective strategies. However, the establishment of gestational age-specific quantitative reference values requires validation in larger cohorts, and long-term follow-up studies are needed to further investigate the correlation between cerebral microcirculation and neurodevelopmental outcomes. In summary, the application of UMA in neonatal intracranial microcirculation assessment holds significant clinical value. It represents a promising advancement in cerebral perfusion monitoring and neurodevelopmental evaluation, particularly for preterm infants, with the potential to improve long-term neurological outcomes.

## Data Availability

The raw data supporting the conclusions of this article will be made available by the authors, without undue reservation.
